# Molecular Dynamics Simulation of Lipid Nanoparticles Encapsulating mRNA

**DOI:** 10.3390/molecules29184409

**Published:** 2024-09-17

**Authors:** Zhigang Zhang, Dazhi Cheng, Wenqin Luo, Donling Hu, Tiantian Yang, Kaixuan Hu, Li Liang, Wei Liu, Jianping Hu

**Affiliations:** 1 Key Laboratory of Medicinal and Edible Plants Resources Development of Sichuan Education Department, School of Pharmacy, Chengdu University, Chengdu 610106, China; 2 School of Life Science, Leshan Normal University, Leshan 614004, China

**Keywords:** mRNA vaccines, lipid nanoparticles (LNPs), encapsulation mechanisms, design, efficacy

## Abstract

mRNA vaccines have shown great potential in responding to emerging infectious diseases, with their efficacy and stability largely dependent on the delivery vehicles—lipid nanoparticles (LNPs). This study aims to explore the mechanisms by which LNPs encapsulate mRNA, as well as the effects of different N/P ratios and acid types in nucleic acid solutions on the structure and properties of LNPs, using the ethanol solvent injection method as the encapsulation technique. Six systems were designed, based on the composition and proportions of the existing mRNA vaccine mRNA-1273, and molecular dynamics (MD) simulations were employed to investigate the self-assembly process of LNPs. Ethanol was used as a solvent instead of pure water to better mimic experimental conditions. The results indicate that lipid components self-assemble into nanoparticles under neutral conditions, with the ionizable lipid SM-102 predominantly concentrating in the core of the particles. Upon mixing with nucleic acids in acidic conditions, LNPs undergo disassembly, during which protonated SM-102 encapsulates mRNA through electrostatic interactions, forming stable hydrogen bonds. Cluster structure analysis revealed that the four lipid components of LNPs are distributed sequentially from the outside inwards as DMG-PEG 2000, DSPC, cholesterol, and protonated SM-102. Moreover, LNPs constructed under low pH or low N/P ratios using citric acid exhibited larger volumes and more uniform distribution. These findings provide a scientific basis for further designing and optimizing LNP components to enhance the efficacy of mRNA vaccine encapsulation.

## 1. Introduction

In recent years, lipid-based drug delivery systems have been widely applied in the field of biomedicine. For instance, lipid nanoparticles (LNPs) are utilized for drug delivery in mRNA-mediated immunotherapy (such as mRNA vaccines) and siRNA-mediated gene silencing therapies, among others [1]. LNPs are typically composed of a mixture of lipids, among which ionizable cationic lipids are considered the most critical components. These ionizable cationic lipids contain positively charged amine groups at low pH, which interact specifically with negatively charged nucleic acids and facilitate membrane fusion during the internalization process [2]. In addition to ionizable cationic lipids, most modern LNPs also include polyethylene glycol (PEG)-lipids, cholesterol, and phospholipids to efficiently encapsulate and deliver mRNA [3].

**Figure 1:**
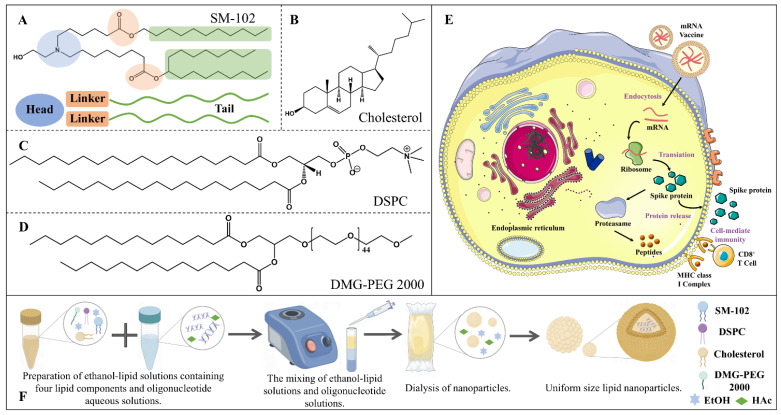
Composition, mechanism of action, and preparation process of mRNA–LNPs. (**A**) Structure of ionizable cationic lipids. (**B**) Structure of cholesterol. (**C**) Structure of phospholipids. (**D**) Structure of polyethylene glycol (PEG). (**E**) Mechanism of action of mRNA vaccines in vivo. (**F**) Preparation process of mRNA–LNPs. mRNA primary sequence: TCGAACGTTCGAACGTTCGAACGTTCGAAT.

The overall structure of ionizable cationic lipids can be divided into three parts: the head group, the linker, and the hydrophobic tail (see Figure 1A) [4]. The head group typically contains a protonatable nitrogen, and its size and charge density are primarily associated with processes such as mRNA encapsulation and interactions with membranes [5]. The linker connects the head group to the tail and mainly affects the stability and transfection efficiency of LNPs. The length and structure of the hydrophobic tail influence the fluidity and fusogenicity of LNPs during membrane fusion [6]. Phospholipid head groups contain negatively charged phosphate groups and positively charged amines, rendering them overall electrically neutral, while their tails consist of long hydrophobic chains (see Figure 1C). Phospholipids are auxiliary lipids that can accelerate mRNA release during endocytosis [7]. Cholesterol, characterized by multiple six-membered rings and a hydroxyl group, primarily mediates LNPs endocytosis, stabilizes the structure of LNPs, and prevents cargo leakage (see Figure 1B) [4]. Polyethylene glycol (PEG)-lipids consist of a backbone of repeating ethylene oxide units, with two hydrophobic tails and a methoxy head group (see Figure 1D). These lipids function by preventing LNP aggregation and fusion, as well as controlling particle size. At high concentrations, they inhibit the fusion of LNPs with cellular membranes, thereby preventing their entry into cells [8]. There have been reports of immune responses triggered by (PEG)-lipids, prompting the search for safer alternatives, such as polysarcosine [9]. Due to their ability to bypass natural barriers and deliver proteins and nucleic acids to biological targets, lipid nanoparticles have attracted significant interest from both the academic and industrial communities.

The COVID-19 pandemic has resulted in millions of deaths worldwide. In December 2020, the U.S. Food and Drug Administration (FDA) and the European Medicines Agency (EMA) approved two mRNA vaccines targeting the spike protein of the severe acute respiratory syndrome coronavirus 2 (SARS-CoV-2), namely BNT162b2 and mRNA-1273 [10]. Both vaccines consist of lipid nanoparticles (LNPs) and nucleoside-modified mRNA encoding the SARS-CoV-2 spike protein. The primary role of LNPs is to protect the mRNA from degradation by nucleases in vivo, thereby enhancing the stability of mRNA in vitro. Upon intramuscular injection, mRNA–LNPs (mRNA lipid nanoparticles) are first taken up by host cells, where the mRNA is subsequently released intracellularly. The mRNA sequence is then translated into the spike protein by ribosomes. Following translation and processing within the host cells, the spike protein appears on the cell surface in a membrane-bound form, providing an antigenic target for B cells. Some of the transiently produced spike proteins enter the antigen presentation pathway, where they are presented via major histocompatibility complex (MHC) molecules to T cells, facilitating T cell antigen recognition (see Figure 1E) [11].

Additionally, vaccine production mainly involves three steps: preparation of lipid and nucleic acid solutions, mixing of lipid and nucleic acid solutions, and storage of mRNA–LNPs. Initially, four lipid components are dissolved in ethanol, based on their molar ratios, and nucleic acids are dissolved in an acidic buffer solution to prepare an mRNA aqueous solution. The lipid–ethanol solution and the nucleic acid aqueous solution are then rapidly mixed to form nanoparticles, typically with an N/P ratio of 3:1 or 6:1 during mixing. Finally, excess lipids and ethanol are removed through methods such as dialysis [12]. Currently, existing mRNA vaccines are preserved for extended periods through freezing, with cryoprotectants such as sucrose and trehalose commonly added to the mRNA–LNPs formulations (see Figure 1F) [13].

The success of COVID-19 vaccines has garnered widespread attention to the study of mRNA–LNPs, with current research focusing primarily on three areas [8]: (i) mRNA sequence design and nucleotide modification, (ii) optimization of LNP formulations, and (iii) long-term storage. Nucleotide modification is considered one of the most significant breakthroughs in the field of mRNA therapeutics, as unmodified messenger RNA can be recognized by cellular RNA sensors, leading to the activation of the innate immune response [14,15], which significantly reduces mRNA translation in host cells [16,17]. In 2005, Karikó et al. discovered that the incorporation of chemically modified nucleosides could significantly reduce the immunogenicity of mRNA [18]. Additionally, some studies have shown that chemical modification of mRNA nucleosides enhances molecular stability and increases protein translation capacity [19,20]. Mauger et al. have demonstrated that naturally occurring modified uridines, such as that obtained when substituting uridine with 1-methyl-pseudouridine, induce global changes in mRNA secondary structure, which is associated with higher protein expression levels [21]. It is worth noting that we believe the method of mRNA encapsulation also deserves significant attention. In many fields, the encapsulation method is crucial for protecting the product from external environmental factors, extending its shelf life, and enabling controlled release [22].

mRNA molecules are highly fragile, necessitating very low storage temperatures for mRNA vaccines to ensure long-term preservation (e.g., Pfizer and BioNTech’s BNT162b2 at −90 to −80 °C, and Moderna’s mRNA-1273 at −25 to −15 °C) [8]. A notable characteristic of mRNA is its long chain, ranging from 1000 to 5000 nucleotides, where even a single change (such as chain breakage or base oxidation) can lead to translation interruption [23]. In vitro degradation of mRNA primarily occurs through hydrolysis and oxidation. Hydrolysis mainly affects the phosphodiester bonds in the mRNA backbone, while oxidation can cause base cleavage, chain breakage, and alterations in the mRNA secondary structure [24]. Hydrolysis is generally considered the primary driver of mRNA degradation [25]. The siRNA-LNPs formulation of Onpattro (Patisaran) has a shelf life of three years at 2–8 °C [26]. Research by Ball et al. using ionizable cationic lipid 306O_13_ as a replacement for DLin-MC3-DMA in the formulation indicates that, under aqueous conditions, the formulation remains stable for 156 days at 2 °C and pH 7, with no significant changes in particle size or siRNA encapsulation efficiency [13]. This suggests that the stability of mRNA, rather than LNPs, determines the storage conditions and shelf life of mRNA–LNP vaccines. The method of lipid encapsulation of nucleic acids not only determines the degree of protection against nucleic acid degradation but also influences how the nucleic acids escape from endosomes following the fusion of nanoparticles with the endosomal membrane. Small-angle neutron scattering (SANS), small-angle X-ray scattering (SAXS), and electron microscopy (EM) experiments reveal that LNPs exhibit varying numbers of layered structures, with lipid bilayers separated by acid-rich regions [27,28,29]. The internal composition of LNPs is predominantly lipid, although some research data suggest the presence of a certain degree of water [27]. This implies that, even when mRNA is encapsulated, it may still be partially exposed to the aqueous environment. This conclusion is supported not only by previous low-temperature transmission electron microscopy results of siRNA-LNPs [30], but also by similar observations of electron-dense cores in mRNA–LNPs [4].

In recent years, although extensive research has been conducted on mRNA–LNPs (lipid nanoparticles), two important scientific issues remain unresolved. For instance, the composition and structure of mRNA–LNPs are still a matter of debate, and the encapsulation mechanism of mRNA is not yet fully understood. Investigating the microscopic dynamics during the preparation of mRNA–LNPs can aid in the optimization of lipid nanoparticle formulations and improve mRNA delivery efficiency. In this study, a simulation system was constructed based on the lipid components and ratios of the mRNA-1273 vaccine [31], and long-term all-atom molecular dynamics (MD) simulations were performed. Using the converged MD trajectories, structural comparisons of mRNA–LNPs were made in two acidic environments (acetic acid and citric acid) and under different (N/P) ratios, employing analyses such as density distribution, radial distribution function, and solvent-accessible surface area (SASA). Subsequently, possible mRNA encapsulation mechanisms were proposed based on binding free energy and cluster analysis. Notably, this study simulates lipid nanoparticles formed through mRNA encapsulation using the solvent injection method, serving as a reference for another experimental study. Overall, this work reveals the molecular recognition and encapsulation processes between mRNA and LNPs. It not only provides guidance for optimizing LNP formulations but also contributes to the precise regulation of LNP stability and mRNA drug release properties, offering significant theoretical and practical value for the development of drug delivery systems.

## 2. Results

### 2.1. Construction of the Investigated Systems

To date, most MD simulation studies of mRNA lipid nanoparticles (LNPs) have used pure water as the simulation environment. For example, Li et al. [32] optimized the lyophilization process of SARS-CoV-2 vaccine mRNA–LNPs by using the transferable intermolecular potential with 3 points (TIP3P) water model to fill the periodic boundary box, with water molecules surrounding the phospholipids and lyophilization protectants. In studies investigating the effect of pH on lipid component distribution, Marius et al. [31] constructed a fully hydrated system based on the lipid composition and ratios of the Comirnaty vaccine and studied the spontaneous aggregation and structural formation of LNPs. In actual mRNA lipid nanoparticle preparation, lipid solutions are often prepared using solvents such as ethanol [33,34] or Dimethyl sulfoxide (DMSO) [35,36]. Moreover, a critical step in the preparation of mRNA lipid nanoparticles is the mixing of lipid components with mRNA in an acidic environment. Previous research has indicated that the type of acid can significantly affect the transfection efficiency of mRNA–LNPs. Cheng et al. [37] noted that LNPs formed in sodium citrate buffer at pH 4 exhibited higher transfection efficiency compared with those formed in sodium acetate buffer at the same pH. Among currently marketed vaccines, the N/P ratios during the mixing of lipid and nucleic acid solutions differ; for instance, the siRNA vaccine Onpattro uses a 3:1 ratio, while mRNA vaccines BNT162b2 and mRNA-1273 use a 6:1 ratio [26].

In this study, 31,408 ethanol molecules were added to each system box to simulate a 300 mg/mL ethanol solution. Two acidic environments were set up using acetic acid (HAc) and citric acid (CA), and two N/P ratios of 3 and 6 were used for mixing lipid and nucleic acid solutions. The pH of two simulated acidic environments was set to 3.4. The amounts of the four lipid components added to the system were based on the following concentrations: SM-102 (34.22 mg/mL), DSPC (7.35 mg/mL), cholesterol (14.34 mg/mL), and DMG-PEG (3.75 mg/mL). Table 1 presents the following six simulation systems: LNP, LNP_HAc, LNP_HAc_DNA, LNP_HAc_2DNA, LNP_CA_DNA, and LNP_CA_2DNA. By comparing LNP and LNP_HAc, the differences in self-assembly of LNPs in neutral versus acidic environments can be investigated. The systems LNP_HAc_DNA and LNP_HAc_2DNA allow for exploration of the structural differences in nanoparticles formed under acetic acid conditions with N/P ratios of 6 and 3, respectively. A comprehensive analysis of LNP_HAc_DNA and LNP_CA_DNA can reveal the impact of acid type on nanoparticle structure formation.

### 2.2. Convergence of MD Trajectories

**Figure 2:**
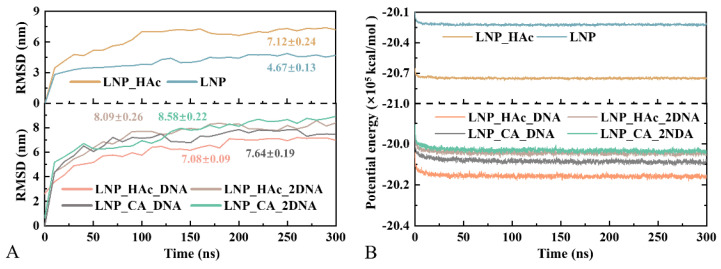
Time evolution of RMSD (**A**) and potential energy (**B**) for the six systems.

Root mean square deviation (RMSD) is used to measure how much a system deviates from its initial structure during simulation and is commonly employed to assess the stability of the system [38]. As shown in Figure 2A, after approximately 200 ns of simulation time, the RMSD values for all systems generally stabilize. Although there are minor fluctuations in each RMSD curve, the overall trend does not show a significant increase or decrease, which is typically considered a sign that the trajectory has converged. Specifically, the LNP_HAc and LNP systems stabilize at around 4.67 ± 0.13 nm and 7.12 ± 0.24 nm, respectively. The higher RMSD value indicates that the lipid components undergo relatively larger structural changes during self-assembly in the acidic environment, possibly involving significant global aggregation and dissociation.

Potential energy is a key indicator of system stability, with lower potential energy generally signifying greater stability [39]. Figure 2B illustrates the changes in potential energy over time for different systems during the MD simulation. After 50 ns, the potential energy curves for all systems stabilize with minimal significant fluctuations, indicating that the potential energy for all systems has converged and the structures are generally approaching dynamic equilibrium.

### 2.3. Macroscopic Characterization of System Dynamics

Snapshots of the six systems were obtained at three time points: 0 ns, 100 ns, and 300 ns. The self-assembly of the four lipid components in acidic and neutral environments showed notable differences in the final conformation at 300 ns. Initially, the four lipid components were uniformly distributed within the box at 0 ns. Over time, they aggregated into small clusters, which then merged to form larger clusters.

**Figure 3:**
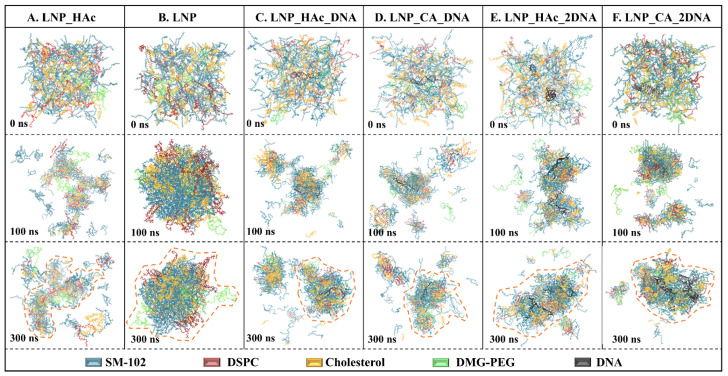
Representative MD simulation snapshots of the six systems: LNP_HAc (**A**), LNP (**B**), LNP_HAc_DNA (**C**), LNP_CA_DNA (**D**), LNP_HAc_2DNA (**E**), and LNP_CA_2DNA (**F**). The largest clusters at the 300 ns snapshot are highlighted with yellow dashed lines.

In the acidic environment, the LNP_HAc system formed numerous small, discrete clusters, with the largest cluster exhibiting a flocculent structure (see Figure 3A). In contrast, the LNP system in the neutral environment formed a single, more regular spherical large cluster (see Figure 3B). This suggests that, in a neutral environment, lipid molecules aggregate into spherical clusters, which, when mixed with acidic nucleic acid solutions, disaggregate into flocculent structures that may facilitate the encapsulation of nucleic acid molecules. To test this hypothesis, a spherical cluster from the neutral environment was subjected to MD simulation in an acidic environment. The resulting snapshots showed that the spherical cluster fragmented during the simulation, supporting the earlier hypothesis (see Figure S1).

Comparing the systems with an N/P ratio of 6:1 in acetic acid and citric acid environments (i.e., LNP_HAc_DNA and LNP_CA_DNA), the encapsulation of nucleic acids and the self-aggregation of lipid components occur simultaneously. Subsequently, the lipid clusters merge with the nucleic acid clusters to form nucleic acid nanoparticles (see Figure 3C,D). In studies of the complexation of single-stranded mRNA with lipids, Rissanou et al. observed, through kinetic snapshots and cluster number analysis, that the self-assembly of clusters in the system coincided with the encapsulation of RNA [40,41].

When multiple nucleic acid strands are present and the N/P ratio is reduced to 3:1 (i.e., in the LNP_HAc_2DNA and LNP_CA_2DNA systems), the nucleic acid clusters merge with each other to form larger nanoparticles. This clearly indicates that multiple nucleic acid strands can be encapsulated within a single lipid nanoparticle, with the size of the nanoparticles being determined by the (PEG)-lipid on the surface [8] (see Figure 3E,F). Interestingly, Gao et al. used computational methods to design optimized siRNA-LNP formulations and found that nanoparticles formed with an N/P ratio of 6 had a larger volume. We speculate that the smaller volume observed in Gao et al.’s study with an N/P ratio of 3 could be due to an insufficient number of lipid components, which may have been a major factor influencing nanoparticle size [42]. Additionally, Jürgens et al. evaluated the properties and efficiency of siRNA and mRNA LNPs, and their particle size data indicate that siRNA-LNPs with an N/P ratio of 3:1 had a larger diameter compared with mRNA–LNPs with an N/P ratio of 6:1 [43], which aligns with our findings.

For the core clusters of the six systems at 300 ns, their radius of gyration (Rg), volume, solvent-accessible surface area (SASA), and density were calculated (see Table 2). The radius of gyration and volume of the LNP_HAc_DNA and LNP_CA_DNA systems were 3.88/4.25 nm and 311.04/402.03 nm^3^, respectively, with densities of 703.20 g/L and 687.03 g/L. This indicates that the nanoparticles formed in the presence of citric acid are larger but more loosely packed, exhibiting a lower density and greater volume. Additionally, the density of the LNP_HAc_2DNA system was 715.76 g/L. When comparing systems with the same acidic environment but different N/P ratios of 3:1 and 6:1 (i.e., LNP_HAc_DNA vs. LNP_HAc_2DNA and LNP_CA_DNA vs. LNP_CA_2DNA), it can be observed that a lower N/P ratio facilitates the formation of larger and denser clusters.

### 2.4. Impact of pH on Lipid Cluster Structure

**Figure 4:**
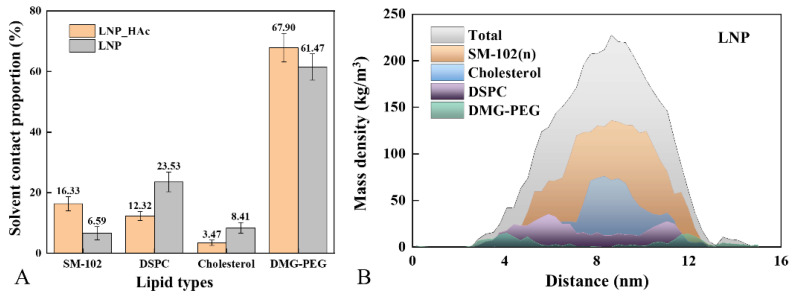
Distribution of lipid components within the core clusters of the systems. (**A**) Average solvent-accessible surface area (SASA) percentage of lipids in the LNP_HAc and LNP systems. (**B**) Density distribution of the lipid core in the LNPs system.

To investigate the distribution differences of the four lipid components within clusters after self-assembly, when lipid solutions transition from a neutral to an acidic environment, the average contact area ratio between the four lipid components and water was measured (see Figure 4A). The results show that, whether in an acidic (LNP_HAc) or neutral (LNP) environment, DMG-PEG always maintains the greatest contact with water, being located on the surface of the lipid clusters [31]. Based on cryogenic transmission electron microscopy (Cro-TEM), small-angle X-ray scattering (SAXS), and small-angle neutron scattering (SANS), Arteta et al. developed a structural model of mRNA–LNPs, indicating that ionizable lipids are primarily located at the core encapsulating the mRNA, while PEG lipids are distributed on the surface of LNPs [27]. Notably, among the other three lipid components, the ionizable lipid SM-102 has the smallest average solvent-accessible surface area ratio of 6.59% in a neutral environment, while in an acidic environment, its ratio is the highest at 16.33%. This suggests that ionizable lipids may undergo a distribution shift when switching between neutral and acidic environments, migrating from the core of the lipid cluster to the surface.

Further density distribution analysis was performed on the spherical clusters of the LNP system (see Figure 4B), revealing that the overall cluster conforms to a normal distribution, forming a dense core and a loose outer shell layer. This finding is consistent with Rissanou et al.’s density analysis of clusters in their study on the complexation of single-stranded RNA with ionizable lipids [41]. The unprotonated ionizable lipid SM-102 and cholesterol are mainly distributed in the dense core region, while DSPC and PEG are located in the hydrophilic outer shell layer. In summary, changes in the solution pH can induce a reversal in the distribution of conformational components, causing ionizable lipids to migrate from the dense core to the hydrophilic surface, thereby disrupting the lipid cluster structure and leading to cluster deconstruction. Marius et al. also suggested this pH-responsive structural change in their study on the phase transition of mRNA lipid nanoparticles [31]. This phenomenon has significant implications in fields such as drug delivery and gene therapy, as it indicates that pH can affect the behavior of lipid nanoparticles, potentially influencing the release efficiency and targeting specificity of drugs or genes.

### 2.5. The Effect of Introducing Different Acids on the Structure of Lipid Clusters

**Figure 5:**
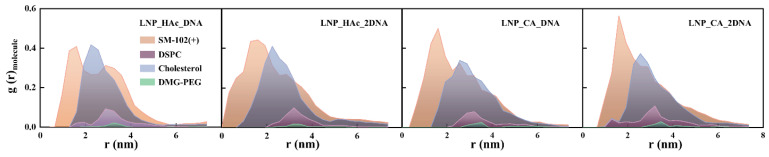
Radial distribution functions of nucleic acids and lipid components in the LNP_HAc_DNA, LNP_HAc_2DNA, LNP_CA_DNA, and LNP_CA_2DNA systems.

Figure 5 shows the radial distribution functions (RDFs) of the four lipid components with nucleic acids in the four mRNA–LNP systems (LNP_HAc_DNA, LNP_HAc_2DNA, LNP_CA_DNA, LNP_CA_2DNA). The results indicate that, in all four systems, the peak positions of the lipid components are in the order of SM-102(+), cholesterol, DSPC, and DMG-PEG, suggesting that the protonated ionizable lipid is closest to the nucleic acid. Previous studies have indicated that under low pH conditions, protonated SM-102 tends to localize at the hydrophilic surface of the nanoparticles. Marketa et al. also found in their study of the spatial distribution of four lipid components in RNA self-assembly systems that ionizable lipids adhere most closely to RNA, while DSPC and PEG are positioned the furthest away [44].

However, during the nucleic acid encapsulation process, protonated SM-102 migrates from the surface of the cluster to the interior of the lipid nanoparticles, encapsulating the nucleic acids, indicating that protonated ionizable lipids play a crucial role in nucleic acid encapsulation. Additionally, the SM-102(+) peak values for the systems LNP_HAc_DNA, LNP_HAc_2DNA, LNP_CA_DNA, and LNP_CA_2DNA are 0.408, 0.442, 0.500, and 0.564, respectively. The radial distribution peaks of ionizable lipids with nucleic acids are higher and more concentrated in citric acid environments, suggesting that citric acid is more favorable for the interaction between ionizable lipids and nucleic acids. Cheng et al. discovered that inducing bubble structures in mRNA lipid nanoparticles can enhance transfection efficiency. In their study comparing acetic acid and citric acid environments when constructing mRNA lipid nanoparticles, they found that nanoparticles formed in a citric acid environment were more conducive to forming bubble structures and had a larger volume, resulting in higher transfection efficiency [37]. This indicates that the acidic environment may not only influence the internal structure of lipid nanoparticles but also affect the strength of interactions between lipids and nucleic acids.

### 2.6. Radial Distribution of Solvent

**Figure 6:**
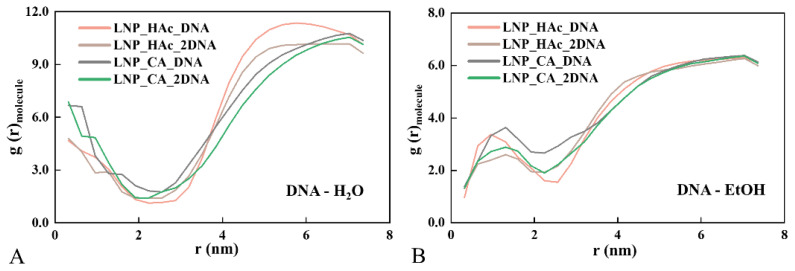
Radial distribution functions of water molecules (**A**) and ethanol molecules (**B**) around nucleic acids in the LNP_HAc_DNA, LNP_HAc_2DNA, LNP_CA_DNA, and LNP_CA_2DNA systems.

To determine the distribution characteristics of solvent molecules within mRNA–LNPs, the radial distribution functions of nucleic acids with water and ethanol molecules were analyzed. The data indicate that the initial structural stacking between nucleic acids and water molecules is strongest, with g values decreasing with increasing distance, reaching a minimum at an r value of 2 nm (see Figure 6A), where it corresponds to the lipid shell predominantly occupied by cholesterol. Conversely, ethanol molecules exhibit lower distribution near the nucleic acids and cholesterol layers, with the highest distribution occurring at r values of 1 nm between the two layers (see Figure 6B). This suggests that there is a solvent cavity between the nucleic acids and the lipid shell, where water molecules are primarily distributed closer to the nucleic acids, while ethanol molecules are more prevalent on the side further from the nucleic acids. This solvent distribution pattern may influence the stability of mRNA–LNPs and their delivery efficiency in vivo by modulating interactions between nucleic acids and lipids.

### 2.7. Dynamic Process of mRNA Encapsulation

**Figure 7:**
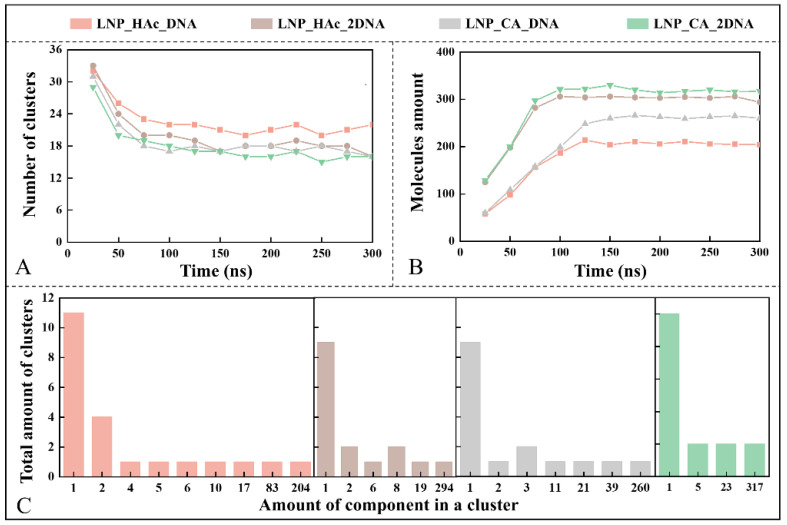
Formation and size distribution of clusters in the LNP_HAc_DNA, LNP_HAc_2DNA, LNP_CA_DNA, and LNP_CA_2DNA systems. (**A**) Variation in the number of clusters over time. (**B**) Number of lipid molecules in the core clusters over time. (**C**) Cluster size and number distribution within the systems at 300 ns.

The changes in cluster formation during the nucleic acid encapsulation process were subsequently investigated. First, the variation in the number of clusters over time was analyzed for the systems LNP_HAc_DNA, LNP_HAc_2DNA, LNP_CA_DNA, and LNP_CA_2DNA (see Figure 7A). The results show that, initially, lipid molecules aggregated to form numerous clusters, which then gradually decreased in number, reaching relative stability at around 100 ns. Figure 7B illustrates the core cluster formation process in the four systems. In both LNP_HAc_DNA and LNP_CA_DNA systems, the number of lipid molecules forming core clusters increased from the start of the simulation and stabilized around 150 ns. Additionally, clusters in the LNP_HAc_2DNA and LNP_CA_2DNA systems stabilized more quickly, reaching relative stability around 100 ns. This indicates that the mRNA encapsulation process is synchronized with self-assembly of LNPs and can be completed in a shorter time. A lower N/P ratio accelerates cluster stabilization. Furthermore, the number of lipid molecules in core clusters formed under citric acid conditions is higher compared with acetic acid, which is consistent with the larger cluster formation observed in the citric acid environment. Figure 7C shows the conformations of the four systems at 300 ns and presents the distribution of cluster number and size. The results reveal that clusters formed in the citric acid environment have a more convergent size distribution, with a lower N/P ratio favoring the formation of larger clusters, while acetic acid environments tend to form smaller clusters with fewer than 10 lipid molecules.

### 2.8. Driving Force of mRNA Encapsulation

**Figure 8:**
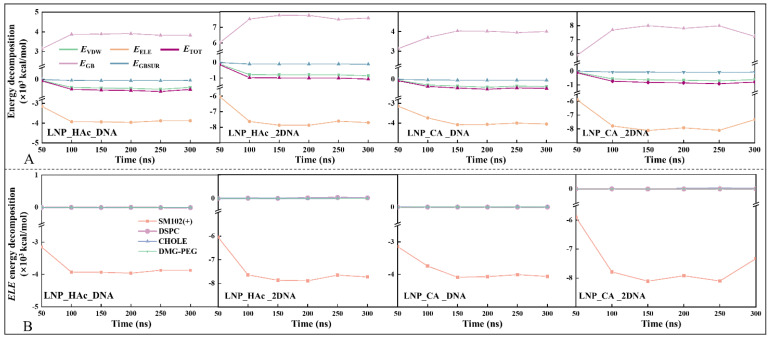
Binding free energy and energy decomposition of nucleic acids and lipids in the LNP_HAc_DNA, LNP_HAc_2DNA, LNP_CA_DNA, and LNP_CA_2DNA systems. (**A**) Decomposition of binding energy components for the four systems. *E*_VDW_ represents van der Waals binding energy under vacuum conditions, *E*_ELE_ represents electrostatic binding energy under vacuum conditions, *E*_GB_ represents polar solvation energy, and *E*_GBSUR_ represents non-polar solvation energy. (**B**) Further decomposition of the electrostatic energy for the four systems.

Binding free energy is a key indicator for evaluating the binding affinity between receptor and ligand. By decomposing the binding free energy into various energy components, one can analyze the contributions of different interactions to the total free energy [45,46], thereby revealing the main driving forces in the nucleic acid encapsulation process. Figure 8A illustrates the decomposition of the total binding energy (*E*_TOT_) into different energy components for the four systems (i.e., LNP_HAc_DNA, LNP_HAc_2DNA, LNP_CA_DNA, and LNP_CA_2DNA). The curves for each energy component stabilize after 100 ns.

For the system LNP_HAc_DNA, the total binding free energy (*E*_TOT_) at 300 ns is −0.50 × 10^3^ kcal/mol, with the individual energy components *E*_VDW_ (van der Waals binding energy), *E*_ELE_ (electrostatic binding energy), *E*_GB_ (polar solvation energy), and *E*_GBSUR_ (non-polar solvation energy) being −0.40 × 10^3^ kcal/mol, −3.88 × 10^3^ kcal/mol, 3.82 × 10^3^ kcal/mol, and −0.05 × 10^3^ kcal/mol, respectively. This indicates that electrostatic interactions in a vacuum environment are the primary driving force for the nucleic acid encapsulation process, while van der Waals forces are more significant in a solvent environment. In other words, *E*_VDW_ primarily provides intermolecular stabilization, while *E*_ELE_ reflects the strong electrostatic interactions between the protonated ionizable lipids and nucleic acids [47]. Further decomposition of the electrostatic energy across the four systems reveals that it is derived mainly from the protonated ionizable lipids (see Figure 8B). Protonated ionizable lipids play a crucial role in the nucleic acid encapsulation process by providing significant electrostatic attraction, thereby stabilizing the nucleic acids within the lipid particles [8].

In summary, during the nucleic acid encapsulation process, electrostatic interactions serve as the primary driving force, while van der Waals forces play a crucial role in maintaining the overall structural stability. These findings not only deepen our understanding of the encapsulation mechanism but also provide theoretical support for the design of more efficient nucleic acid delivery systems.

### 2.9. Stability Factor of mRNA–LNPs

**Figure 9:**
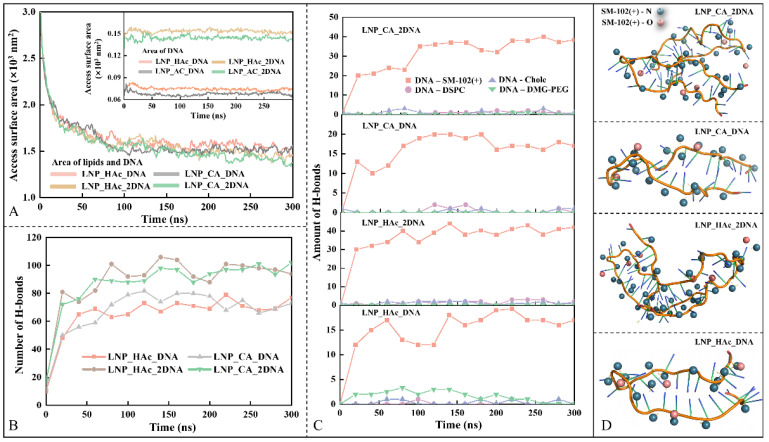
Changes in solvent-accessible surface area (SASA) and hydrogen bond counts over time for nucleic acids and lipids in the LNP_HAc_DNA, LNP_HAc_2DNA, LNP_CA_DNA, and LNP_CA_2DNA systems. (**A**) Solvent-accessible surface area (SASA) as a function of time. (**B**) Total number of hydrogen bonds within the system over time. (**C**) Number of hydrogen bonds between lipid components and nucleic acids over time. (**D**) Three-dimensional visualization of hydrogen bonds between nucleic acids and ionizable lipids, with blue and red spheres representing nitrogen and oxygen atoms of the ionizable lipids, respectively.

Figure 9A shows the solvent-accessible surface area (SASA) of lipid components and nucleic acids in the LNP_HAc_DNA, LNP_HAc_2DNA, LNP_CA_DNA, and LNP_CA_2DNA systems over time. Initially, the SASA for all four systems decreases rapidly and stabilizes around 150 ns, indicating that the encapsulation process is largely complete. Concurrently with the encapsulation of nucleic acids, the total number of hydrogen bonds in the four systems also stabilizes around 150 ns (see Figure 9B), consistent with the SASA results. In this study, hydrogen bonds were identified using geometric criteria: the distance between the hydrogen atom and the receptor atom is less than 0.35 nm, and the angle formed by the receptor atom–hydrogen atom–ligand atom is greater than 135° [48]. As shown in Figure 9C, hydrogen bonds between ionizable lipids and nucleic acids constitute the majority of the total hydrogen bonds in the system, with hydrogen bonds formed between ionizable lipids and nucleic acids primarily provided by protonated nitrogen (see Figure 9D).

Integrating the analysis of driving forces and hydrogen bonding, we found that during the formation of mRNA–LNPs, electrostatic forces drive the lipid components to encapsulate the mRNA. Subsequently, hydrogen bonds between the protonated nitrogen and oxygen atoms of ionizable lipids and the mRNA stabilize the encapsulation of mRNA.

## 3. Discussion

In this study, we discuss the effects of different N/P ratios on the encapsulation of mRNA by lipid components and whether the type of acid used during the preparation of mRNA–LNPs plays a significant role. Additionally, we explore the mechanism of mRNA encapsulation and the structure of mRNA–LNPs. In the discussion section of this paper, we compare current research on mRNA vaccines with our study through Table 3.

The structure of mRNA lipid nanoparticles (LNPs) has long been a subject of debate, particularly regarding the presence of internal solvent cavities. Our study demonstrates the formation of solvent cavities between the lipid layers and mRNA, encapsulating the mRNA. The choice of N/P ratio, which is a critical aspect in the formulation of mRNA vaccines, has been widely discussed. We found that an N/P ratio of 6 promotes better fusion between clusters when compared with a ratio of 3, although this conclusion remains controversial. The choice of acidic solvent for preparing the nucleic acid solution also affects the formation of lipid nanoparticles. Our findings align with those of Cheng et al., who have also observed that mRNA–LNPs formed with citric acid are larger [37]. However, we have discovered that LNPs formed with acetic acid are more compact. Regarding the mechanism of mRNA encapsulation, the prevailing view is that ionizable lipids play a crucial role. We propose that ionizable lipids encapsulate mRNA through electrostatic interactions driven by protonation, leading to a redistribution of components.

With the rapid development of mRNA delivery systems, exploring optimized N/P ratios and more appropriate acidic solvent choices will be key directions for the further improvement of the performance of mRNA–LNPs. Additionally, combining molecular dynamics simulations with experimental techniques could reveal more details about the internal microstructure of nanoparticles, paving the way for breakthrough advancements in mRNA vaccines.

## 4. Calculation Methods

### 4.1. System Preparation

Using the mRNA-1273 vaccine as a reference, four lipid mixture configurations (SM-102:DSPC:Cholesterol:DMG-PEG 2000 = 50:10:38.5:1.5) were constructed at pH 4 and pH 7 to study lipid self-assembly and mRNA encapsulation. Environments with pH 4 were created using either acetic acid (HAc) or citric acid (CA), with the amount of ionized/non-ionized molecules of acetic acid or citric acid determined based on their pKa values, resulting in 2/33 or 22/12, respectively. Additionally, all lipid amines were selected for protonation while keeping other lipid components unchanged. Given that the simulation environment is of an ethanol solution, 20,918 ethanol molecules were added to each box. For the study of LNP self-assembly, the numbers of SM-102, DSPC, cholesterol, and DMG-PEG 2000 molecules in the system were 144, 30, 111, and 4, respectively. For mRNA encapsulation studies, the numbers of these molecules were 174, 35, 134, and 5, respectively.

The mRNA used in this study is TCGAACGTTCGAACGTTCGAACGTTCGAAT, with a secondary structure primarily characterized by {…(((((((((((....)))))))))))…}. Based on the primary sequence and secondary structure, the 3D structure of the mRNA was constructed using the 3D-RNA/DNA web server (http://biophy.hust.edu.cn/new/3dRNA) [49]. Nucleotide parameters were derived from the CHARMM36 force field [50,51]. Notably, this server calculates the 3D structure by searching for 3D templates from experimental structures, assembling the structure based on each smallest secondary element (SSE) with similarity to the target sequence, and then performing Amber energy minimization to avoid atomic clashes. This server has been widely used. For instance, Mu et al. used the 3D-RNA/DNA server to build 3D structures of single-stranded and double-stranded DNA in their study of stability predictions, comparing these structures with experimental data and showing low RMSD values [52]. Cao et al. utilized the 3D-RNA/DNA server to construct the structure of circular RNA (circMYLK4) and simulate its binding with the CACNA2D2 protein [53].

### 4.2. Molecular Dynamics Simulation

The topology and various parameters for atomic interactions and intra-atomic interactions of cholesterol and DSPC were obtained from the CHARMM36 force field [54,55]. Because no available topology files existed for SM-102 and DMG-PEG 2000, the Sobtop tool (http://sobereva.com/soft/Sobtop, accessed on 3 October 2023) was used to generate topology files for these molecules based on their coordinate files. All systems underwent energy minimization using the steepest descent algorithm, with convergence criteria set to a difference in energy between adjacent conformations of less than 1000 kJ·mol^−1^·nm^−1^, or until a maximum of 20,000 steps was reached.

In the molecular dynamics (MD) simulations, a Nose–Hoover thermostat [56,57] was used to maintain constant temperature, with a temperature coupling constant of 1.0 ps. During system preparation, the Berendsen barostat [58] was chosen for its high numerical stability, with a reference pressure of 1 bar, a pressure coupling constant of 5.0 ps, and a compressibility of 4.5 × 10^−5^ bar^−1^. During the MD simulations, the pressure was regulated using the Parrinello–Rahman barostat [59], which better represents the NPT ensemble. Van der Waals interactions were managed using a force-based switch function (1.0–1.2 nm), while long-range Coulombic interactions were handled using the particle mesh Ewald method [60]. The LINCS algorithm [61] was used to constrain bonds between heavy atoms and hydrogen, with a LINCS order of 4 per iteration step. The simulations employed a time step of 2 fs, with a reference temperature of 310 K and a box size of 20 × 20 × 20 nm^3^, using the CHARMM TIP3P [62] water model. All system preparations, trajectory generation, and result analyses were performed using the GROMACS software package (v2019.3) [63].

### 4.3. Spatial Density Distribution

The lipid composition of the monolayer and core of the LNPs was determined by fitting a spherical grid to the LNP surface. The grid points were placed on a unit sphere using a spiral method [64], and the calculation formula is as follows [31]:

(1) $$\theta_{a}=arcos\left(\frac{2a-1-A}{A}\right)\phi_{a}=\sqrt{\pi A}\times arcsin\left(\frac{2a-1-A}{A}\right)$$

where *a* = 1,…, *A*, with *A* representing the total number of grid points. In this work, a balance between speed and accuracy was achieved by setting *A* to 750. After constructing the grid, each point was iteratively displaced along the inward normal of the sphere. The iteration stopped when a grid point came within 1.5 nm of a lipid atom. Subsequently, interpolation was performed by adding midpoints between adjacent grid points, specifically for all points within a distance of 4.0 nm. Lipids within 2.5 nm of the grid were assumed to be part of the surrounding monolayer. The core composition was then calculated by subtracting the number of lipids in the LNP shell from the total number of lipids in the simulated system.

### 4.4. Radial Distribution Function

Based on the MD simulation trajectories, the radial distribution function (RDF) is used to analyze the spatial distribution of atoms or molecules under given conditions, thereby providing insights into the interactions between particles in solutions or solids. In the simulation system, the space is divided into small cubic grid points. For a specific atom or molecule, spherical shells with a given radius range are created around it. For each central particle, the number of particles in each spherical shell is counted and then normalized to account for volume effects. This process yields the particle density around the central particle at different radial ranges. By comparing the normalized particle density with the volume of the spherical shells, the average density of particles within each shell is obtained. These density values form the RDF, which describes the average distribution of particles at different distances. The RDF between type A and type B particles, also known as the pair correlation function $g\left(\gamma \right)$, is defined as follows [41]:

(2) $$g\left(\gamma \right)=\frac{1}{\rho N}\frac{dN\left(r\right)}{4\pi r^{2}dr}$$

where ρ represents the average particle density of the system, N is the total number of particles in the system, $N\left(r\right)$ denotes the number of particles within a shell of thickness dr at a distance r from the reference particle, and dr is a small increment in the shell thickness.

### 4.5. Binding Free Energy Calculation

Binding free energy is a crucial physicochemical parameter commonly used to evaluate molecular recognition between receptors and ligands, and it is also a key standard for assessing the activity of drug molecules [65]. In this study, the binding free energy between mRNA and the lipid components in the system was predicted using the molecular mechanics/Poisson–Boltzmann surface area (MM/PBSA) algorithm [45], which was used to assess the driving forces for mRNA encapsulation. In MM/PBSA, the binding free energy is decomposed into different energy terms. All energy terms are calculated based on a large number of conformations generated from GROMACS simulations; thus, longer MD simulation times and more extensive sampling result in more accurate statistical values for these energy terms. The binding free energy, Δ*G*_bind_ can be expressed as follows [66]:

(3) $$\Delta G_{\mathrm{bind}}=E_{MM}-Τ\Delta S+\Delta G_{solv}$$

Here, solvent water is excluded from the MD trajectories to obtain solute conformations as a function of time, and the total internal energy $E_{MM}$ is computed under the molecular mechanics force field. Τ is the absolute temperature in Kelvin, and ΔS represents the change in conformational entropy calculated using the normal mode approach, which primarily characterizes the change in conformational disorder within the complex. The solvation free energy $\Delta G_{solv}$ can be divided into electrostatic and non-polar interactions; the electrostatic component is generally obtained by solving the Poisson–Boltzmann equation using finite difference methods, while the contribution of the non-polar component is calculated using the solvent-accessible surface area (SASA).

## 5. Conclusions

Based on comparative MD simulations of six systems (LNP, LNP_HAc, LNP_HAc_DNA, LNP_HAc_2DNA, LNP_CA_DNA, and LNP_CA_2DNA), the structural characteristics of mRNA–LNPs have been detailed, shedding light on the potential mechanisms for mRNA encapsulation in lipid nanoparticles (LNPs) used in mRNA vaccines. This study specifically analyzes the effects of different N/P ratios and types of acidic environments on the formation of mRNA nanoparticles. The findings indicate that, under neutral conditions, lipid components self-assemble into nanoparticles, with ionizable lipids predominantly residing in the core of the lipid nanoparticles. When mixed with nucleic acids in an acidic environment, ionizable lipids undergo protonation, causing a redistribution of lipids that leads to the disintegration of the nanoparticles. The electrostatic interactions drive the encapsulation of mRNA, while protonated nitrogen atoms in ionizable lipids form stable hydrogen bonds with mRNA, ensuring the structural stability of the mRNA–LNP complexes. Radial distribution analysis of the mRNA–LNP components reveals that the distribution of the four lipid components within the nanoparticles follows a sequence from the outer layer to the core, as follows: DMG-PEG 2000, DSPC, cholesterol, and SM-102 (protonated). Additionally, mRNA–LNP clusters formed at lower N/P ratios exhibit larger volumes, higher densities, and greater uniformity. The type of acidic environment also affects the formation of mRNA–LNP clusters; citric acid environments are more favorable for forming larger, more uniform clusters with a looser internal structure compared to acetic acid.

In summary, the simulation results provide valuable insights for the design and optimization of mRNA–LNPs formulations and for enhancing vaccine efficacy.

## Figures and Tables

**Table 1:** Description of the six simulated systems.

Components												
Systems	SM-102(+)	SM-102(n.)	DSPC	Cholesterol	DMG-PEG	HAc	Ac^−^	CA	CA^−^	EtOH	DNA	N/P
LNP	0	144	30	111	4	0	0	0	0	31,408	0	0
LNP_HAc	144	0	30	111	4	33	2	0	0	31,408	0	0
LNP_HAc_DNA	174	0	35	134	5	33	2	0	0	31,408	1	6:1
LNP_HAc_2DNA	174	0	35	134	5	33	2	0	0	31,408	2	3:1
LNP_CA_DNA	174	0	35	134	5	0	0	12	22	31,408	1	6:1
LNP_CA_2DNA	174	0	35	134	5	0	0	12	22	31,408	2	3:1

**Table 2:** Characterization parameters of key clusters in simulation systems.

Systems	Components						Rg (nm)	Volume (nm^3^)	SASA (nm^2^)	Density (g/L)
	SM-102(+)	SM-102(n.)	DSPC	Cholesterol	DMG-PEG	DNA				
LNP	0	144	30	111	4	0	3.42	238.08	609.91	717.50
LNP_HAc	144	0	30	111	4	0	5.10	290.99	874.40	795.04
LNP_HAc_DNA	174	0	35	134	5	1	3.88	311.04	817.75	703.20
LNP_HAc_2DNA	174	0	35	134	5	2	4.34	446.93	1112.78	715.76
LNP_CA_DNA	174	0	35	134	5	1	4.25	402.03	1091.92	687.03
LNP_CA_2DNA	174	0	35	134	5	2	4.72	479.61	1285.55	700.02

**Table 3:** Comparison and differences of various aspects of the study with the literature.

Aspects	Literature	Comparison and Differences
Structure of mRNA–LNPs	Schoenmaker, L., et al., 2021 [26].	The presence of internal solvent cavities
N/P	Gao, H., et al., 2022 [42]. Jürgens, D.C., et al., 2023 [43].	The effect of N/P ratio on cluster fusibility
Types of acidic environments	Cheng, M.H.Y., et al., 2023 [37].	It was consistently found that citric acid is more conducive than acetic acid to the formation of larger lipid nanoparticles
Mechanism of mRNA encapsulation	Trollmann, M.F., et al., 2022 [31].	It was consistently found that the key to the mRNA encapsulation process lies in the redistribution of ionizable lipids in response to pH changes

## Data Availability

All data generated or analyzed during this study are included in this published article.

## Supplementary Materials

The following supporting information can be downloaded at: https://www.mdpi.com/article/10.3390/molecules29184409/s1.
