# Supplementary figures and images for: Molecular Dynamics Simulation of Lipid Nanoparticles Encapsulating mRNA
Source: Molecules. 2024 Sep 17;29(18):4409. doi: 10.3390/molecules29184409 (PMC11433737; doi:10.3390/molecules29184409)

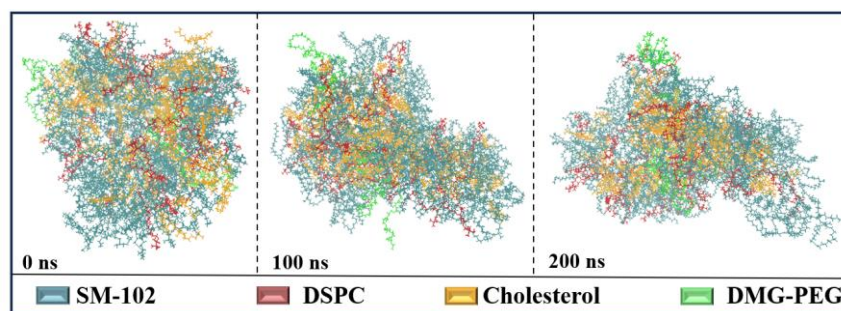

**Figure S1.** Kinetic snapshots of lipid nanoparticles in an acidic environment.

Supplement: Supplementary file 1 [file molecules-29-04409-s001.zip › molecules-3199969-supplementary.pdf]
